# Supplementary material for: Comparative Cutaneous Water Loss and Desiccation Tolerance of Four Solenopsis spp. (Hymenoptera: Formicidae) in the Southeastern United States
Source: Insects. 2020 Jul 5;11(7):418. doi: 10.3390/insects11070418 (PMC7412113; doi:10.3390/insects11070418)

Supplementary Material

Table S1. Relationship between desiccation time (h) at 30°C and 0-2%RH and % initial mass loss by live small, medium, and large workers of *S. richteri*, *S. invicta* × *S. richteri*, *S. invicta*, *S. geminata* ants (mean ± SE). The significance level is 0.05 (Tukey HSD tests).

Species	Stage	N	a ± SE	b ± SE	r^2	Df	F Values	*p* Values
S. richteri	Small	15	72.96 ± 3.20	2.76 ± 0.42	0.9895	6	473.22	<0.0001
	Medium	14	74.33 ± 2.03	3.73 ± 0.31	0.9966	6	1476.28	<0.0001
	Large	15	81.85 ± 3.34	8.38 ± 0.74	0.9961	6	1284.09	<0.0001
S. invicta ×	Small	15	75.44 ± 4.10	2.98 ± 0.55	0.9850	6	329.03	<0.0001
S. richteri	Medium	15	80.31 ± 4.14	6.37 ± 0.79	0.9924	6	654.83	<0.0001
	Large	15	96.33 ± 5.49	13.61 ± 1.44	0.9954	6	1083.48	<0.0001
S. invicta	Small	15	79.99 ± 4.68	4.17 ± 0.70	0.9866	6	367.07	<0.0001
	Medium	15	83.94 ± 4.76	7.81 ± 0.99	0.9922	6	636.57	<0.0001
	Large	14	107.90 ± 11.30	20.26 ± 3.51	0.9905	6	520.30	<0.0001
S. geminata	Small	15	70.62 ± 3.04	2.60 ± 0.40	0.9896	6	474.02	<0.0001
	Medium	15	79.03 ± 3.66	7.17 ± 0.77	0.9942	6	854.19	<0.0001
	Large	15	101.54 ± 10.89	18.92 ± 3.42	0.9890	6	449.62	<0.0001

a = the maximum asymptotic value of % initial mass loss; b = the period required for half the maximum value to be reached.

Table S2. Relationship between desiccation time (h) at 30°C and 0-2%RH and %TBW lost by live small, medium, and large workers of *S. richteri*, *S. invicta* × *S. richteri*, *S. invicta*, *S. geminata* ants (mean ± SE). The significance level is 0.05 (Tukey HSD tests).

Species	Stage	N	a ± SE	b ± SE	r^2	Df	F Values	*p* Values
S. richteri	Small	15	108.05 ± 4.68	2.76 ± 0.42	0.9898	6	486.79	<0.0001
	Medium	14	113.77 ± 3.19	4.05 ± 0.33	0.9967	6	1499.02	<0.0001
	Large	15	131.26 ± 5.33	8.47 ± 0.75	0.9962	6	1310.94	<0.0001
S. invicta ×	Small	15	107.01 ± 6.18	3.14 ± 0.60	0.9837	6	302.43	<0.0001
S. richteri	Medium	15	123.26 ± 6.39	6.41 ± 0.80	0.9924	6	650.27	<0.0001
	Large	15	150.98 ± 8.72	13.63 ± 1.46	0.9953	6	1055.96	<0.0001
S. invicta	Small	15	110.90 ± 6.68	4.25 ± 0.73	0.9860	6	351.11	<0.0001
	Medium	15	127.13 ± 7.09	7.78 ± 0.97	0.9924	6	655.62	<0.0001
	Large	14	167.94 ± 17.66	20.21 ± 3.52	0.9904	6	514.03	<0.0001
S. geminata	Small	15	100.82 ± 4.54	2.64 ± 0.43	0.9888	6	440.63	<0.0001
	Medium	15	121.52 ± 5.56	7.08 ± 0.75	0.9943	6	866.32	<0.0001
	Large	15	161.05 ± 17.08	18.55 ± 3.33	0.9890	6	447.94	<0.0001

a = the maximum asymptotic value of % TBW lost; b = the period required for half the maximum value to be reached.

Table S3. Relationship between desiccation time (h) at 30°C and 0-2%RH and % initial mass loss by dead small, medium, and large workers of *S. richteri*, *S. invicta* × *S. richteri*, *S. invicta*, *S. geminata* ants (mean ± SE). The significance level is 0.05 (Tukey HSD tests).

Species	Stage	N	a ± SE	b ± SE	r^2	Df	F Values	*p* Values
S. richteri	Small	15	67.22 ± 1.47	2.08 ± 0.18	0.9969	6	1586.20	<0.0001
	Medium	15	70.59 ± 3.33	3.56 ± 0.52	0.9900	6	494.94	<0.0001
	Large	15	77.63 ± 6.51	6.95 ± 1.36	0.9820	6	273.24	<0.0001
S. invicta ×	Small	15	70.23 ± 2.91	1.89 ± 0.33	0.9884	6	424.35	<0.0001
S. richteri	Medium	15	73.30 ± 3.37	2.74 ± 0.44	0.9886	6	435.28	<0.0001
	Large	15	75.86 ± 4.93	4.88 ± 0.86	0.9853	6	335.54	<0.0001
S. invicta	Small	15	73.71 ± 2.79	2.29 ± 0.33	0.9912	6	566.19	<0.0001
	Medium	15	72.89 ± 4.28	4.03 ± 0.69	0.9861	6	354.17	<0.0001
	Large	14	79.50 ± 7.11	8.49 ± 1.65	0.9829	6	288.05	<0.0001
S. geminata	Small	15	67.70 ± 1.92	1.72 ± 0.22	0.9942	6	850.63	<0.0001
	Medium	15	75.59 ± 5.02	6.01 ± 0.99	0.9870	6	381.06	<0.0001
	Large	15	97.21 ± 10.13	16.19 ± 2.98	0.9884	6	425.71	<0.0001

a = the maximum asymptotic value of % initial mass loss; b = the period required for half the maximum value to be reached.

Table S4. Relationship between desiccation time (h) at 30°C and 0-2%RH and %TBW loss by dead small, medium, and large workers of *S. richteri*, *S. invicta* × *S. richteri*, *S. invicta*, *S. geminata* ants (mean ± SE). The significance level is 0.05 (Tukey HSD tests).

Species	Stage	N	a ± SE	b ± SE	r^2	Df	F Values	*p* Values
S. richteri	Small	15	112.99 ± 2.37	2.03 ± 0.17	0.9971	6	1707.51	<0.0001
	Medium	15	121.06 ± 5.38	3.46 ± 0.48	0.9909	6	545.19	<0.0001
	Large	15	132.39 ± 10.78	6.81 ± 1.31	0.9827	6	283.82	<0.0001
S. invicta ×	Small	15	116.77 ± 5.06	1.92 ± 0.35	0.9874	6	392.31	<0.0001
S. richteri	Medium	15	120.23 ± 5.55	2.77 ± 0.45	0.9886	6	434.45	<0.0001
	Large	15	122.97 ± 8.01	4.87 ± 0.86	0.9852	6	333.73	<0.0001
S. invicta	Small	15	117.12 ± 4.50	2.27 ± 0.34	0.9909	6	546.91	<0.0001
	Medium	15	124.96 ± 7.33	4.07 ± 0.70	0.9862	6	357.96	<0.0001
	Large	14	131.21 ± 11.79	8.64 ± 1.68	0.9831	6	290.85	<0.0001
S. geminata	Small	15	109.81 ± 3.16	1.62 ± 0.22	0.9938	6	802.42	<0.0001
	Medium	15	123.96 ± 8.34	6.00 ± 1.00	0.9867	6	371.49	<0.0001
	Large	15	158.00 ± 16.15	16.15 ± 2.91	0.9888	6	440.98	<0.0001

a = the maximum asymptotic value of % TBW lost; b = the period required for half the maximum value to be reached.

Figure S1. Mean (± SE) percentage of total body water (%TBW) for: (A) Live small, medium, and large fire ant worker size-classes compared within each *Solenopsis richteri* (Black), *S. invicta* × *S. richteri* (Hybrid), *S. invicta* (Red), and *S. geminata* (Geminata) fire ant species; and (B) Dead small, medium, and large fire ant worker size-classes compared within each *Solenopsis* species. Means with the same letter within each worker size-class or species are not significantly different ($P < 0.05$). N = 14 or 15 individuals per worker size-class per species.

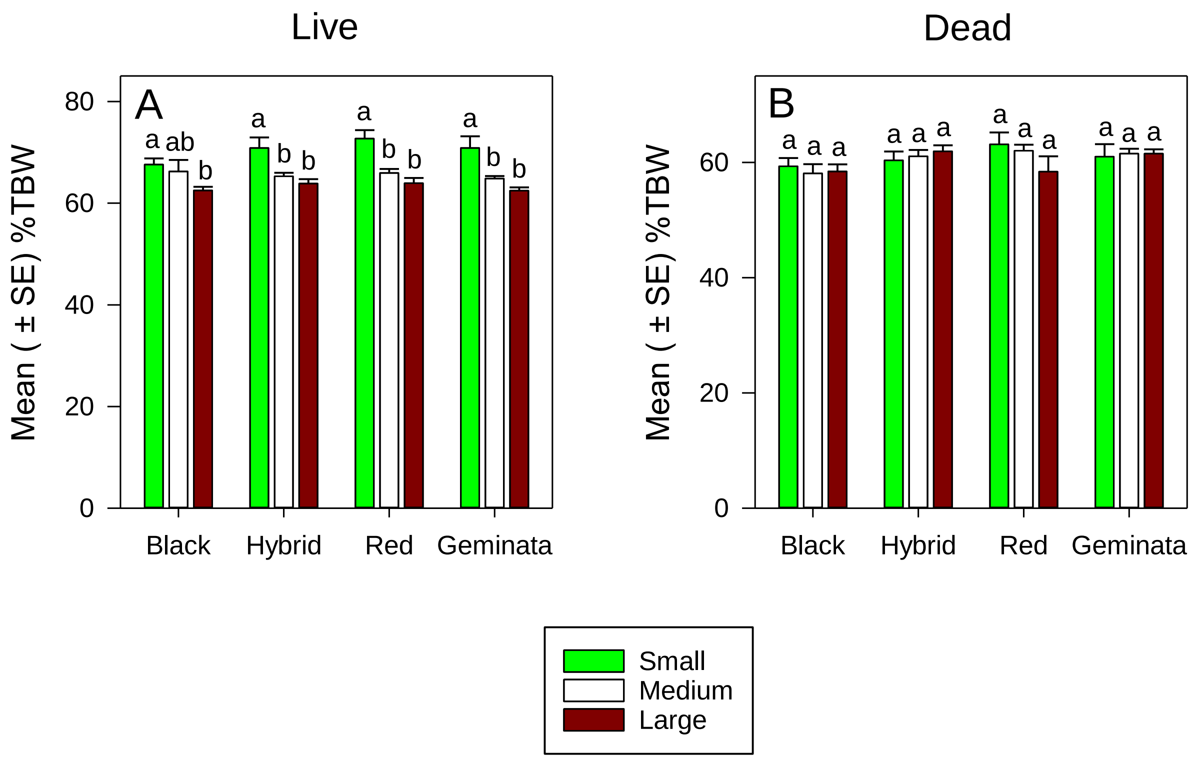

Figure S2. Mean (± SE) adjusted mass loss for: (A) Dead small, medium, and large fire ant worker size-classes compared within each *Solenopsis richteri* (Black), *S. invicta* × *S. richteri* (Hybrid), *S. invicta* (Red), and *S. geminata* (Geminata) fire ant species; and (B) Live small, medium, and large fire ant worker size-classes compared within each *Solenopsis* species. Means with the same letter within each worker size-class or species are not significantly different ($P < 0.05$). N = 14 or 15 individuals per worker size-class per species.

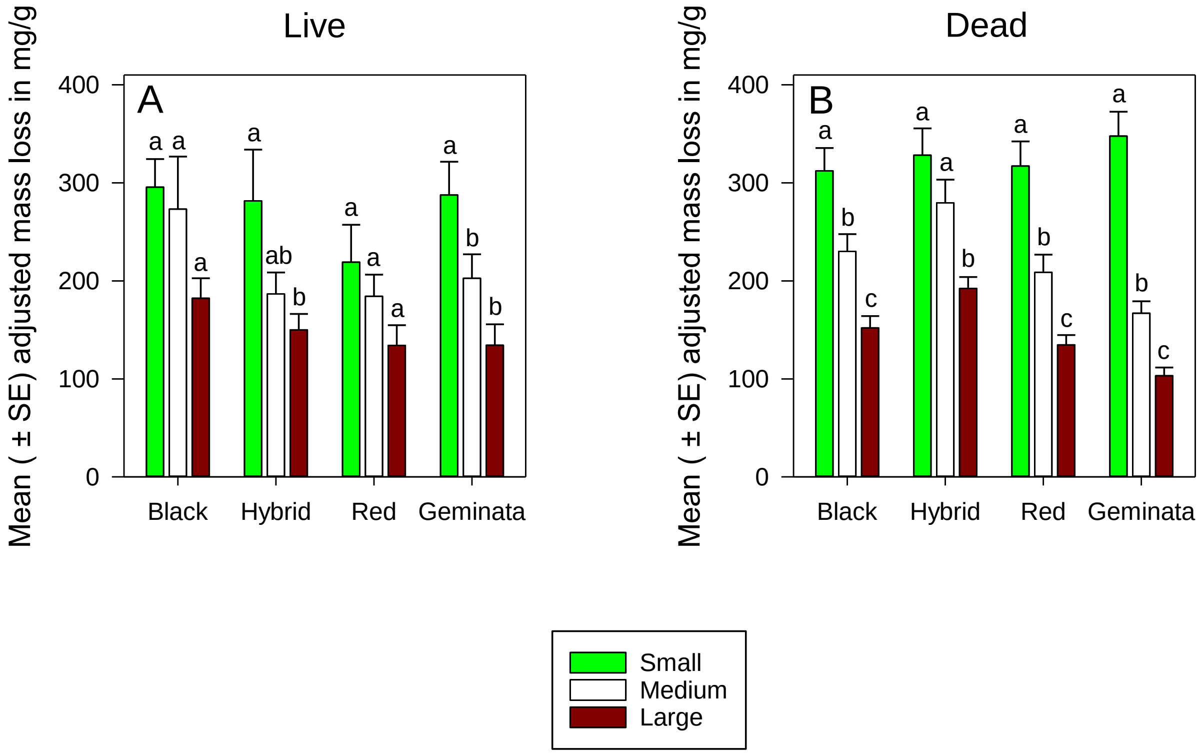

Figure S3. Percentage of initial mass loss over time for live small, medium, and large workers of: (A) *S. richteri* (Black); (B) *S. invicta* × *S. richteri* (Hybrid); (C) *S. invicta* (Red); and (D) *S. geminata* (Geminata). N = 14 or 15 individuals per worker size-class per species.

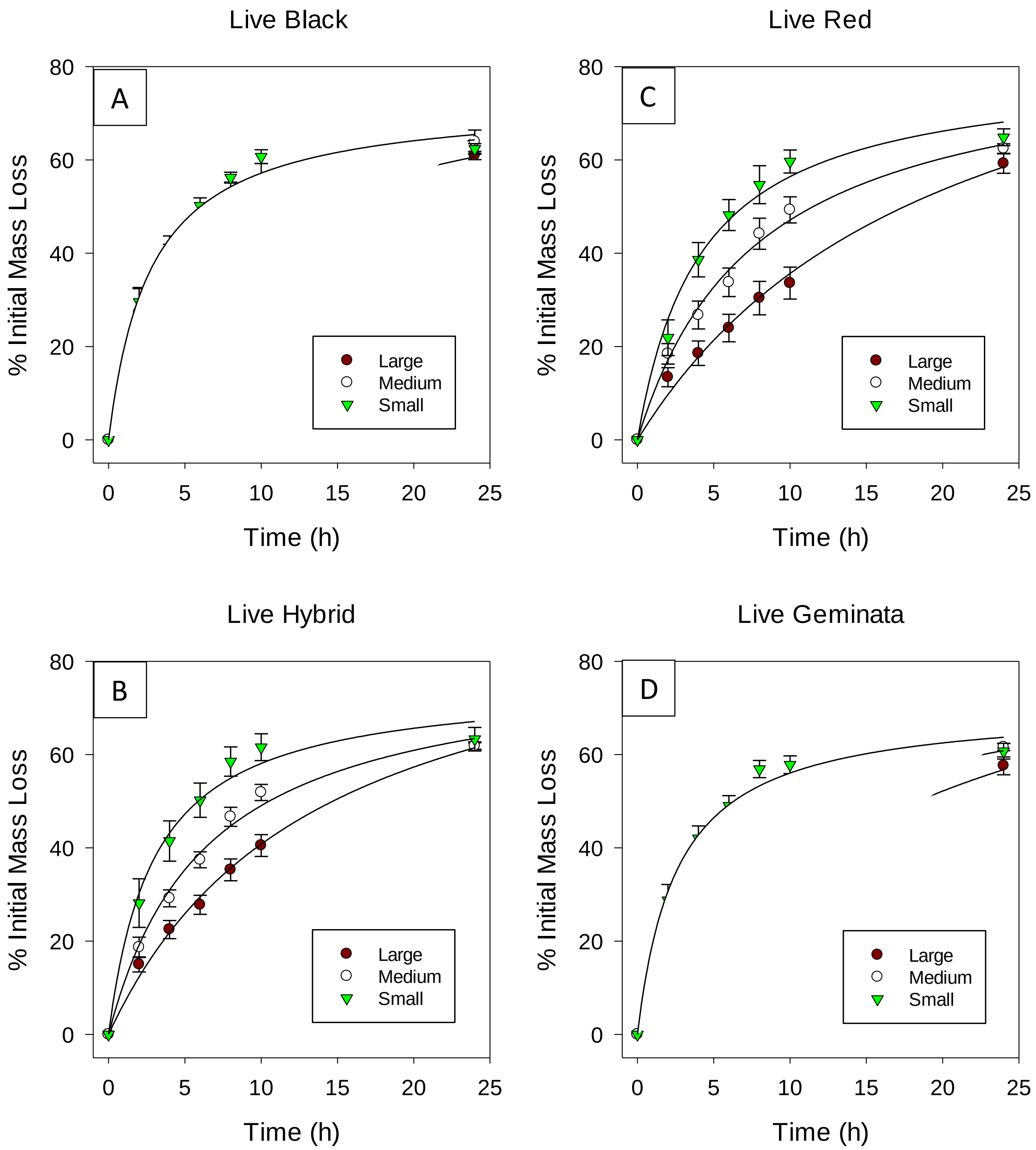

Figure S4. Percentage of total body water (%TBW) lost over time for live small, medium, and large workers of: (A) *S. richteri* (Black); (B) *S. invicta* × *S. richteri* (Hybrid); (C) *S. invicta* (Red); and (D) *S. geminata* (Geminata). N = 14 or 15 individuals per worker size-class per species.

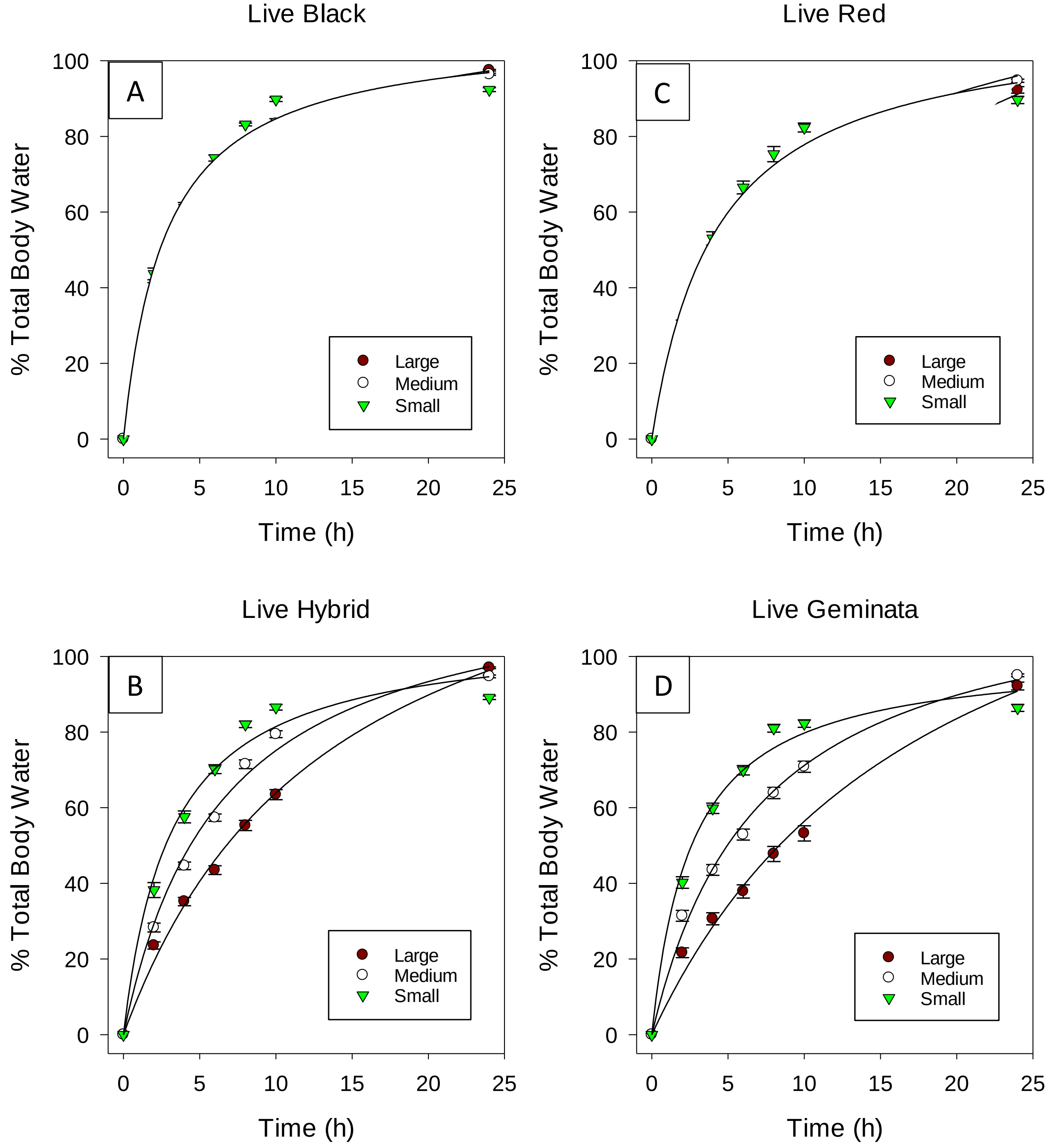

Figure S5. Percentage of initial mass loss over time for dead small, medium, and large workers of: (A) *S. richteri* (Black); (B) *S. invicta* × *S. richteri* (Hybrid); (C) *S. invicta* (Red); and (D) *S. geminata* (Geminata). N = 14 or 15 individuals per worker size-class per species.

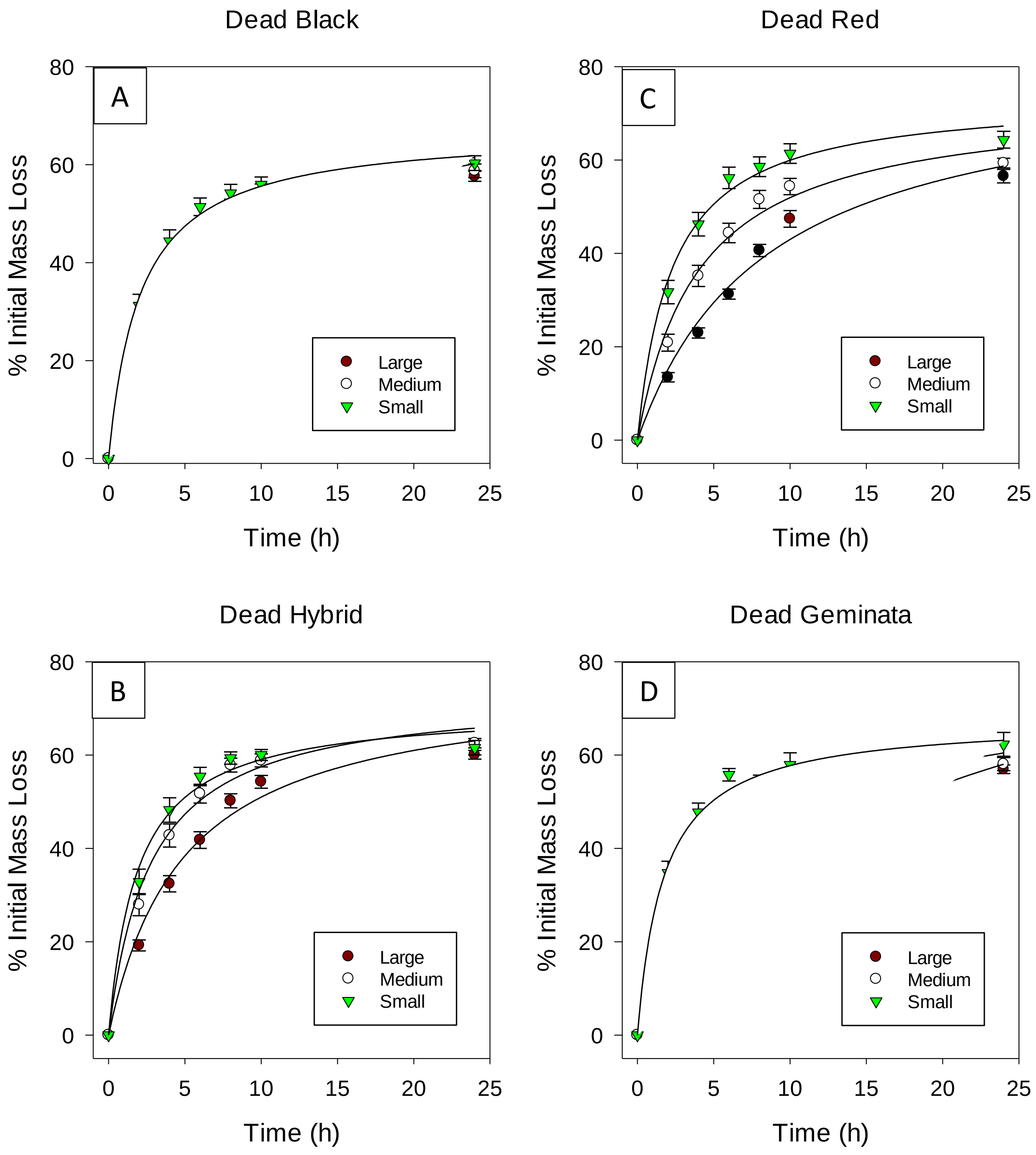

Figure S6. Percentage of total body water (%TBW) lost over time for dead small, medium, and large workers of: (A) *S. richteri* (Black); (B) *S. invicta* × *S. richteri* (Hybrid); (C) *S. invicta* (Red); and (D) *S. geminata* (Geminata). N = 14 or 15 individuals per worker size-class per species.

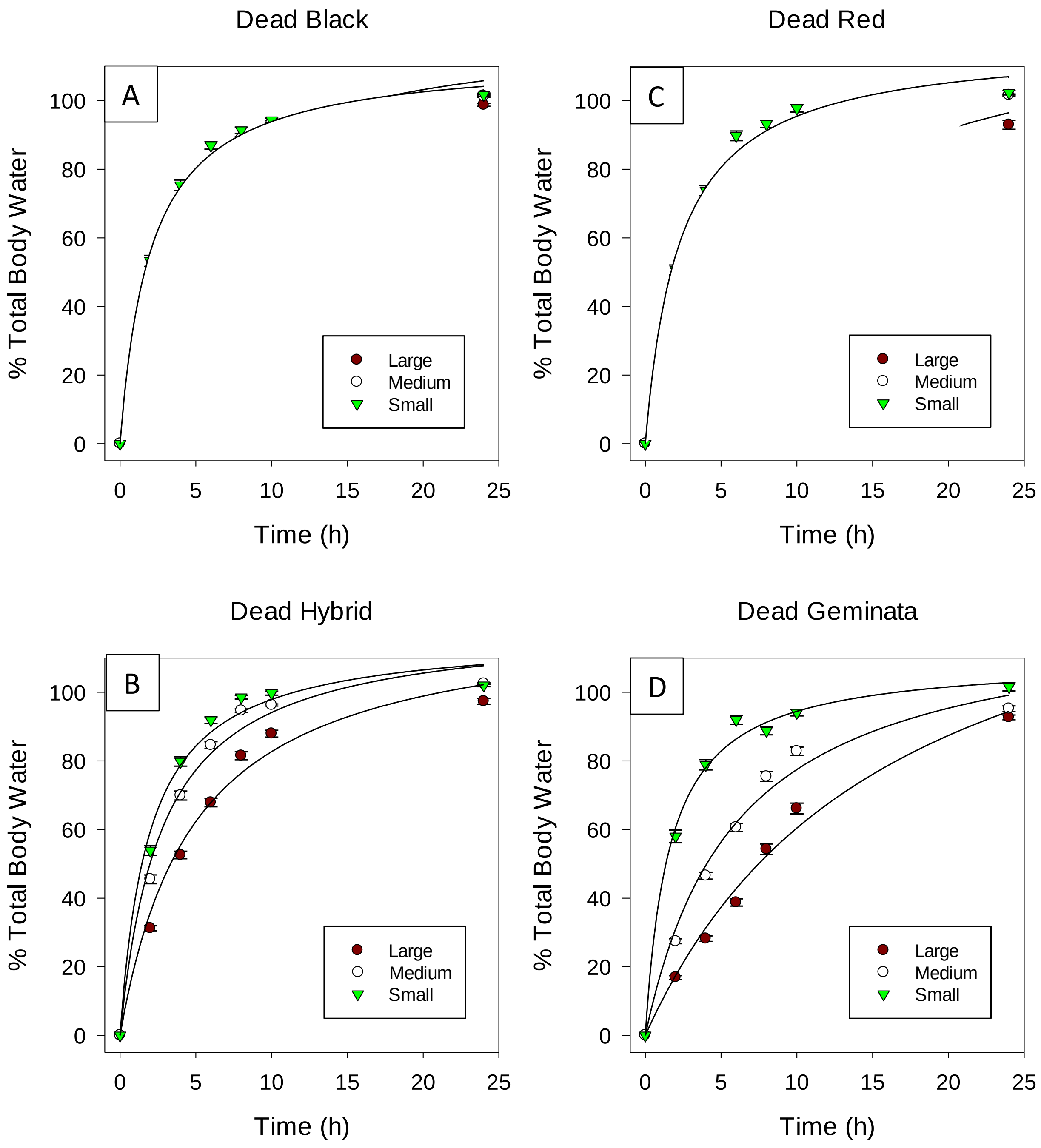

Supplement: Supplementary file 1 [file insects-11-00418-s001.pdf]
